# Ozenoxacin 1% in Pediatric and Adult Patients with Impetigo: A Meta-Analysis of Randomized Trials

**DOI:** 10.3390/jcm14072157

**Published:** 2025-03-21

**Authors:** Bryam López Tuesta, Camila A. Arones-Santayana, Gustavo A. Valderrama Sáenz, Yerson Alberca-Naira, Hector I. Cedillo Torres, Abdias Baltazar Castellanos, Daniel B. Müller Quintanilla, Judith Yangali-Vicente, Joshuan J. Barboza

**Affiliations:** 1 Hospital Nacional Edgardo Rebagliati Martins, Lima 15072, Peru; blt138@hotmail.com; 2 Centro de Investigación en Atención Primaria de Salud, Universidad Peruana Cayetano Heredia, Lima 15102, Peru; camila.arones@upch.pe; 3 Facultad de Medicina, Universidad Privada Antenor Orrego, Trujillo 13008, Peru; gvaldsaenz@gmail.com (G.A.V.S.); dbrayan98@hotmail.com (D.B.M.Q.); 4 Escuela de Medicina, Universidad Nacional de Piura, Piura 20002, Peru; yerson210918@gmail.com; 5 Hospital General Dr. Manuel Gea Gonzalez, Ciudad de Mexico 14080, Mexico; drcedillo2003@gmail.com; 6 Escuela de Enfermeria, Universidad Autónoma del Estado de México, Valle de Chalco 56615, Mexico; abdias.bcg@hotmail.com; 7 Direccion de investigacion, Universidad Inca Garcilazo de la Vega, Lima 15046, Peru; judith.yangali@uigv.edu.pe; 8 Escuela de Medicina, Universidad Señor de Sipan, Chiclayo 14000, Peru

**Keywords:** impetigo, children, adolescents, adults, ozenoxacin

## Abstract

**Introduction**: Impetigo is a relatively common superficial infection of the skin and soft tissues. Although its prevalence is more significant in childhood, it might also occur in adulthood, affecting the quality of life of our patients. **Methods**: A systematic review and meta-analysis of randomized controlled trials (RCTs) comparing ozenoxacin 1% with placebo or mupirocin was conducted. Databases searched included PubMed, Embase, Cochrane Library, and ClinicalTrials.gov. Pooled risk ratios (RRs) with 95% confidence intervals (CIs) were estimated using a random-effects model, and heterogeneity was assessed using I^2^ statistics. **Results**: Four RCTs with 754 patients met the inclusion criteria. Ozenoxacin significantly improved the clinical success (RR: 1.14, 95% CI: 1.04–1.26, and I^2^ = 0%) and reduced the clinical failure (RR: 0.54, 95% CI: 0.39–0.75, and I^2^ = 0%) compared to the placebo. Microbiological success was also superior (RR: 1.31, 95% CI: 1.05–1.58, and I^2^ = 4%), while the microbiological failure was significantly lower (RR: 0.31, 95% CI: 0.21–0.46, and I^2^ = 0%). Comparisons with mupirocin showed similar efficacy, though the estimates were less precise. **Conclusions**: Ozenoxacin 1% is an effective treatment for impetigo, significantly improving clinical and microbiological outcomes while reducing the failure rates compared to the placebo. Its efficacy is comparable to mupirocin, suggesting it as a viable alternative for first-line therapy. Given the low heterogeneity observed, these findings support the clinical use of ozenoxacin for impetigo management. Future large-scale RCTs and direct comparative studies are warranted to further validate its therapeutic benefits.

## 1. Introduction

Impetigo is among the most common bacterial skin infections in adolescents and children [1], especially those under five. However, there is a notable difference in prevalence: 12.3% of cases occur in children, while only 4.9% occur in adults. Ozenoxacin is a novel, non-fluorinated quinolone antibiotic that exerts its bactericidal activity by inhibiting bacterial DNA gyrase and topoisomerase IV, enzymes which are essential for DNA replication and transcription. Unlike other topical antibiotics, such as mupirocin and fusidic acid, ozenoxacin has demonstrated low resistance development and potent activity against common impetigo-causing pathogens, particularly *Staphylococcus aureus* (including methicillin-resistant strains) and *Streptococcus pyogenes*. These attributes make it a promising alternative in the treatment of impetigo, particularly in settings where antimicrobial resistance to traditional treatments is increasing. Moreover, its favorable safety profile and rapid bactericidal action provide advantages for both pediatric and adult populations [2].

Impetigo is classified as a primary bacterial infection, in which bacteria directly invade healthy skin, or as a secondary infection [3], also called common impetigo, which arises in association with another skin condition, such as scabies or eczema [4]. There are two main types of impetigo: non-blister impetigo, which accounts for 70% of cases, and bullous impetigo, which accounts for 30%. Non-blister impetigo, also known as contagious impetigo, is caused by *Staphylococcus aureus* or *Streptococcus pyogenes* [5]. In contrast, bullous impetigo, caused by a toxin-producing strain of *S. aureus*, is characterized by the appearance of flaccid blisters that proliferate and may rupture, ooze, and leave a distinctive collar of scales, and may be caused by a variety of bacteria [6].

Factors that increase the risk of developing impetigo include living in tropical climates, overcrowded conditions, poor hygiene, socioeconomic deprivation, compromised immune status, and participation in close-contact sports [7]. The diagnosis of this pathology is clinical, so it is essential to identify the lesions correctly. It is characterized by coalescent vesicles that leave myoelectric crusts when ruptured and dry. Bullous impetigo presents flaccid vesicular lesions that, when ruptured, generate an eroded surface [8].

Treatment varies according to the extent and location of the lesions, as well as the presence of systemic symptoms. Practical clinical recommendations suggest topical antibiotics for localized lesions and systemic oral antibiotics when topical therapy fails, or when there are systemic complications [9]. Increasing antimicrobial resistance to topical mupirocin and fusidic acid has had adverse consequences for individuals and communities [10], necessitating the development of new treatment alternatives to promote the judicious use of existing medications [11]. Ozenoxacin, a quinolone, received approval in May 2019 by the European Medicines Agency (EMA) in 12 EU (European Union) countries to treat non-blistering impetigo in patients six months and older. In contrast, in the United States and Canada, the Food and Drug Administration (FDA) approved ozenoxacin 1% cream for the treatment of impetigo in patients two months of age and older [12].

Recognizing the importance and prevalence of this pathology, this systematic review and meta-analysis seeks to examine the evidence on the effectiveness of ozenoxacin 1% to reduce the burden of impetigo in endemic and non-endemic settings in the population.

## 2. Materials and Methods

### 2.1. Searches

References were identified through PubMed, Scopus, Web of Science, and Embase for papers published. Searches were performed from the inception until 8 June 2024. The terms included critical phrases, MESH (PubMed), and Emtree thesauri (Scopus and Embase). Finally, a search strategy was applied to each database (Supplementary Table S1). (“Child OR Adolescent OR Adult”) AND (impetigo) AND (Ozenoxacin) were the main keywords used in the research. There were no limitations on the language or date of publication. The gray literature was not included in this study.

### 2.2. Eligibility Criteria

Inclusion Criteria: We included all studies that meet the following criteria: Randomized controlled trials, phase II or III, that included all patients (child and adults) diagnosed with bullous or non-bullous impetigo and assessed the effect of ozenoxacin 1% applied twice daily for 5 to 7 days, compared to standard treatment or placebo. These documents were collected in full text without language preferences or date of publication. The search was carried out from the inception until 8 June 2024.

Exclusion criteria: Systematic reviews, narrative reviews, case reports, series, letters to the editor, conference abstracts, articles without full texts, and other in vitro and in vivo studies will be excluded.

### 2.3. Outcomes

The primary outcomes were the clinical success and failure of the ozenoxacin treatment on bullous and non-bullous impetigo (measured by the relative risk). The secondary outcomes were the microbiological success and failure (measured by the relative risk).

### 2.4. Selection and Data Extraction

The results were compiled following the electronic searches into a single library, with duplicates removed. The initial screening step was conducted, where titles and abstracts were evaluated, and inclusion and exclusion criteria were applied using the Rayyan Web platform. Studies that passed this phase were full-text searches and analyses, followed by a secondary screening process to justify the inclusion and exclusion criteria. The studies assessed eligible after this process were included in the systematic review, and data extraction was started. A third review author (J.J.B.) was consulted in case of any disagreement.

Data were extracted from each study individually and blinded using a pre-prepared Excel spreadsheet format. For each analysis, data were extracted on the author, year of publication, country, type of study, number of participants per intervention arm, selection criteria, description of intervention and control arm, and primary and secondary outcomes.

### 2.5. Risk of Bias Assessment

The risk of bias (RoB) was independently evaluated using the RoB 2.0 tool. Any disagreements were resolved through discussion with a third author (J.J.B.). The RoB for each domain and study was categorized as low, with some concerns, or high for randomized controlled trials (RCTs).

### 2.6. Data Synthesis

All meta-analyses of the effects of ozenoxacin compared to standard treatment on primary and secondary outcomes applied random-effects models with the inverse variance method. The between-study tau^2^ variance was calculated using the Paule–Mandel method. The effects of ozenoxacin compared to the placebo on the dichotomous outcomes were expressed as relative risks (RRs) with 95% confidence intervals (CIs). We applied the continuity correction method for randomized controlled trials (RCTs) with null events in one or both arms. If more than five studies were included in the meta-analysis, the Hartung–Knapp method was used for adjustment. The statistical heterogeneity among RCTs was assessed using the I^2^ statistic, with values indicating low (<30%), moderate (30–60%), and high (>60%) levels of heterogeneity. Sensitivity analyses were conducted using fixed effects and the Mantel–Haenszel method. The metabin function from the R 3.5.1 Meta library (www.r-project.org, accessed on 3 February 2025) was employed. Publication bias was evaluated using a funnel plot and Egger’s test.

### 2.7. GRADE Assessment

The GRADE methodology assessed the evidence’s certainty and the intervention’s degree of recommendation regarding all outcomes [13]. GRADE is based on its domains, such as risk of bias, inconsistency, indirectness, imprecision, and publication bias, which are some of the criteria that will be evaluated. The certainty of the evidence was determined by the outcome and described in the summary of results (SoF) tables, which were created using the online tool GRADEpro GDT (Supplementary Table S2).

## 3. Results

### 3.1. Selection of Studies

**Figure 1:**
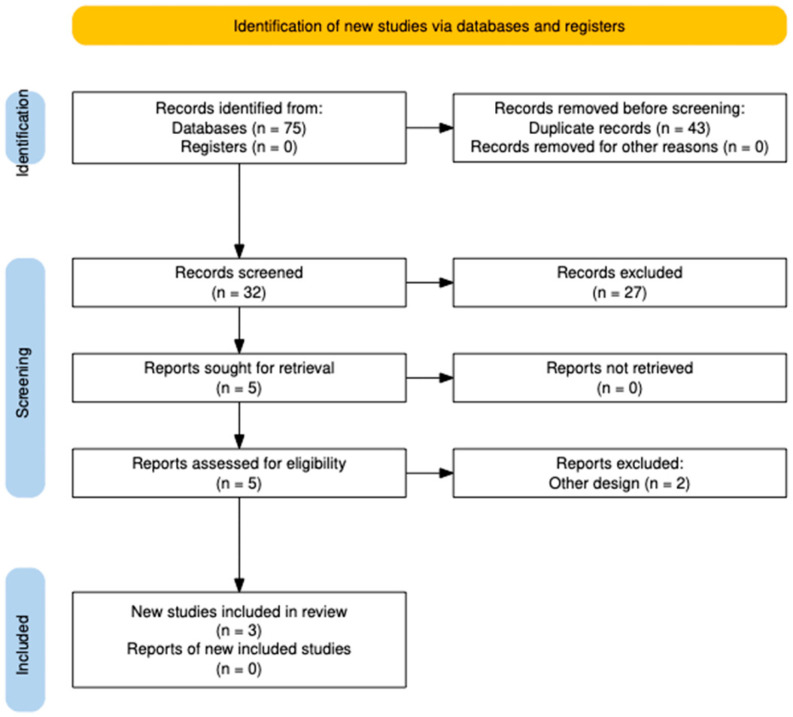
PRISMA 2020 [16]: Flowchart of the selection of studies.

A total of 75 studies were identified through PubMed (n = 19), Scopus (n = 27), Web of Science (n = 11), and Embase (n = 18). After removing duplicates, a total of 32 studies remained. After screening, a total of five studies remained. Two studies were excluded due to methodological limitations: one was an observational study rather than a randomized controlled trial (RCT), and another did not provide sufficient data to assess the effect of ozenoxacin. Finally, three studies were included in this systematic review and meta-analysis [4,14,15] (Figure 1).

### 3.2. Characteristics of Included Studies

The three included studies were phase 3 randomized controlled clinical trials and parallel designs; two were multicenter studies, and one was a single-center study. They were conducted in South Africa, Germany, Romania, Ukraine, Spain, the United States, and India. The studies included 377 patients in the experimental group and 379 in the control group.

Regarding the intervention, in the three studies, ozenoxacin 1% cream was applied topically twice daily to patients with a clinical diagnosis of impetigo. The duration of the treatment varied from 5 to 7 days. In two studies, the control was a placebo, and, in one study, mupirocin was applied (Table 1).

### 3.3. Risk of Bias Assessment

**Figure 2:**
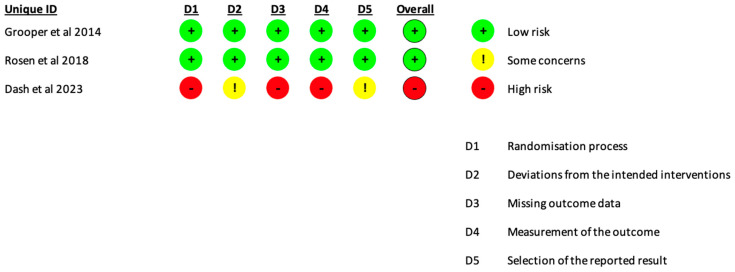
RoB 2.0 for bias assessment [4,14,15].

In one of the three studies evaluated, there was a high risk of bias in randomization process, domain 1, missing outcome data, domain 3, and a measurement of the outcome, domain 4. The other studies obtained a low overall risk of bias (Figure 2).

### 3.4. Effects of Ozenoxacin 1% in Primary and Secondary Outcomes

In this meta-analysis evaluating the efficacy of ozenoxacin 1% in pediatric and adult patients with impetigo, the following four primary outcomes were analyzed: clinical success, clinical failure, microbiological success, and microbiological failure.

**Figure 3:**
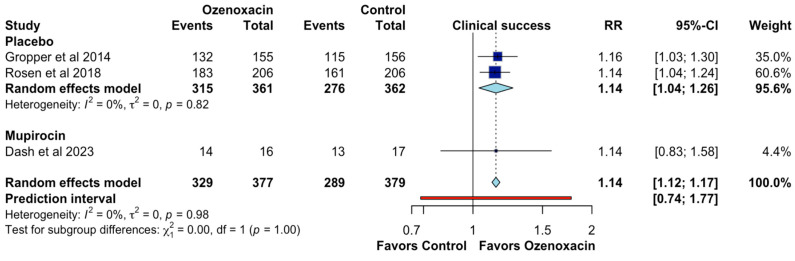
Effects of ozenoxacin 1% on the clinical success [4,14,15].

For clinical success, data from three studies were included, comparing ozenoxacin against placebo and mupirocin. The pooled analysis showed that ozenoxacin significantly improved the clinical success rates compared to the control interventions, with a random-effects model risk ratio (RR) of 1.14 (95% CI: 1.04–1.26; QoE, moderate certainty; Figure 3). Subgroup analysis revealed a similar effect when compared with the placebo (RR: 1.14 and 95% CI: 1.04–1.24) and mupirocin (RR: 1.14 and 95% CI: 0.83–1.58). The heterogeneity was minimal (I^2^ = 0%), indicating consistency across the included studies. The prediction interval [0.74–1.77] suggests that future studies may yield varying results, but the overall effect remains in favor of ozenoxacin.

**Figure 4:**
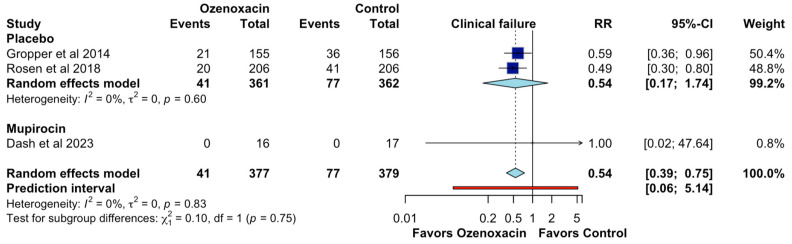
Effects of ozenoxacin 1% on the clinical failure [4,14,15].

Regarding the clinical failure, the pooled analysis indicated that ozenoxacin significantly reduced the clinical failure rates compared to the placebo, with a combined RR of 0.54 (95% CI: 0.39–0.75; CoE, moderate certainty; Figure 4), suggesting a 46% reduction in the treatment failure risk. The effect was more pronounced in individual studies, with RR estimates of 0.59 (95% CI: 0.36–0.96; CoE, moderate certainty; Figure 4) and 0.49 (95% CI: 0.30–0.80). No clinical failure events were reported in the mupirocin subgroup, leading to an RR of 1.00 (95% CI: 0.02–47.64). Heterogeneity was low (I^2^ = 0%), reinforcing the robustness of the findings. The prediction interval [0.06–5.14] suggests that variability in future studies may influence the effect size.

**Figure 5:**
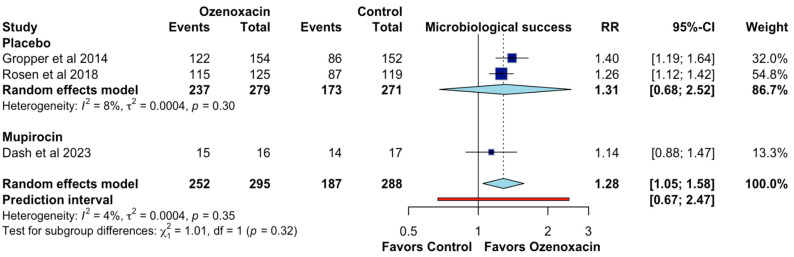
Effects of ozenoxacin 1% on the microbiological success [4,14,15].

For the microbiological success, ozenoxacin demonstrated a higher eradication rate of bacterial pathogens than the placebo, with a pooled RR of 1.31 (95% CI: 1.05–1.58; CoE, moderate certainty; Figure 5), indicating a 31% improvement. Individual study estimates ranged from RR 1.40 (95% CI: 1.19–1.64) to RR 1.26 (95% CI: 1.12–1.42). The comparison with mupirocin showed a similar trend (RR: 1.14 and 95% CI: 0.88–1.47). Heterogeneity was minimal (I^2^ = 4%), indicating consistent results across studies. The prediction interval [0.67–2.47] suggests that, while the overall effect is in favor of ozenoxacin, future studies may present some variability.

**Figure 6:**
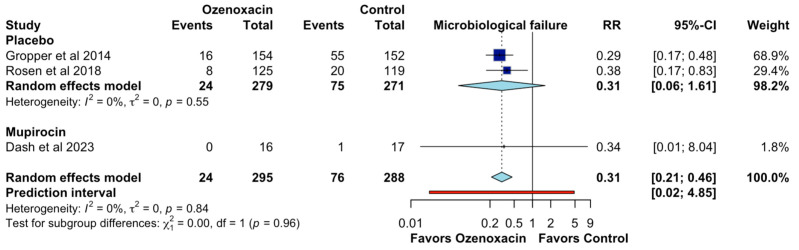
Effects of ozenoxacin 1% on the microbiological failure [4,14,15].

Finally, for the microbiological failure, the pooled RR of 0.31 (95% CI: 0.21–0.46) indicated that ozenoxacin significantly reduced the microbiological failure compared to the control treatments. Individual study estimates showed a reduction in the microbiological failure with RRs of 0.29 (95% CI: 0.17–0.48; CoE, moderate certainty; Figure 6) and 0.38 (95% CI: 0.17–0.83) for the placebo-controlled studies. The comparison with mupirocin yielded an RR of 0.34 (95% CI: 0.01–8.04), though this estimate was imprecise due to the small sample size. Heterogeneity was low (I^2^ = 0%), and the prediction interval [0.02–4.85] suggested some variability in future research outcomes.

In summary, the findings indicate that ozenoxacin 1% is significantly more effective than the placebo in achieving clinical and microbiological success, while reducing the failure rates. The comparison with mupirocin suggests comparable efficacy, although some estimates are imprecise due to the limited number of studies. Given the low heterogeneity across the analyses, these results support the use of ozenoxacin as an effective treatment for impetigo. Future studies with larger sample sizes and direct head-to-head comparisons with standard treatments will be valuable to confirm these findings.

#### Adverse Events

Among the three included studies, the overall incidence of adverse events was low. The most commonly reported events were mild local skin reactions, including erythema (redness) and pruritus (itching), which resolved without medical intervention. No severe adverse events or systemic side effects were reported in any of the included trials. The safety profile of ozenoxacin was comparable to mupirocin, with no significant differences in the frequency or severity of adverse effects.

## 4. Discussion

In the present systematic review and meta-analysis, we evaluated the clinical and microbiological efficacy of ozenoxacin 1% for treating localized impetigo in pediatric and adult patients. The results reveal that ozenoxacin 1% effectively resolves the signs and symptoms of impetigo and provides an effective microbiological cure.

Our main findings are based on the clinical and bacteriological success of ozenoxacin. Regarding the clinical success, we found that ozenoxacin 1% significantly increases the clinical success compared to standard treatment, with an RR of 1.14 (95% CI 1.12 to 1.17), accompanied by a low heterogeneity (I^2^ = 0%). A more significant decrease in clinical failure was also observed, with an RR of 0.54 (95% CI 0.39 to 0.75; I^2^ = 0%). On the other hand, regarding the microbiological success, a better response was observed with the use of ozenoxacin, with an RR of 1.28 (95% CI 1.05 to 1.58; I^2^ = 4%) and an improvement in the microbiological failure of lesions, with an RR 0.31 (95% CI 0.21 to 0.46; I^2^ = 0%). The observed efficacy could be due to its potent bactericidal action against *Staphylococcus aureus* and *Streptococcus pyogenes*, the primary pathogens responsible for impetigo, eventually decreasing the risk of systemic complications [5,17].

The increasing resistance to topical antibiotics, particularly mupirocin and fusidic acid, has raised concerns about the long-term efficacy of standard treatments for impetigo. Ozenoxacin, a novel non-fluorinated quinolone, has demonstrated a low resistance potential in vitro and in clinical studies. Unlike fluoroquinolones, which are prone to mutations in DNA gyrase (gyrA and gyrB) and topoisomerase IV (parC and parE), ozenoxacin has shown minimal cross-resistance with other quinolones. Furthermore, its limited systemic absorption reduces the likelihood of inducing resistance in commensal flora. However, continuous post-marketing surveillance is necessary to monitor the resistance patterns, especially with increasing clinical use [13,14].

Regarding the dosage and duration, clinical trials have established that ozenoxacin 1% cream should be applied twice daily for a duration of 5 to 7 days. Studies indicate that five days of treatment is sufficient for clinical and microbiological resolution in most cases, with no additional benefit observed with prolonged use. The maximum recommended amount is up to 100 cm^2^ of affected skin per application, covering localized lesions without exceeding the safety thresholds. This regimen ensures optimal efficacy while minimizing the risk of bacterial adaptation and resistance development [14].

Previous studies suggest a superiority of ozenoxacin 1% over placebo and other topical antibiotics, such as mupirocin, especially in scenarios of increasing bacterial resistance. Grooper et al. [14] demonstrated that ozenoxacin was superior to the placebo (success rate of 34.8 vs. 19.2%; *p* = 0.003). They also obtained a microbiological success rate of 70.8% for ozenoxacin and 38.2% for the placebo after 3–4 days, and 79.2% vs. 56.6% after 6–7 days.

Rosen et al. [4] demonstrated similar results; clinical success was superior compared to the placebo (112 of 206 [54.4%] vs. 78 of 206 [37.9%]; *p* = 0.001). Ozenoxacin also demonstrated superior microbiologic success compared with the placebo after two days of therapy (109 of 125 [87.2%] vs. 76 of 119 [63.9%]; *p* = 0.002). Dash et al. [15] reported superior clinical (14 of 16 vs. 13 of 17) and microbiological (15 of 16 vs. 14 of 17) success compared with mupirocin after seven days of therapy.

Increasing mupirocin resistance has been documented in multiple countries, with resistance rates reaching 11–20% in some regions. In these settings, the effectiveness of standard topical treatments may be compromised, leading to higher treatment failure rates.

Ozenoxacin has demonstrated a low resistance potential, retaining activity against quinolone-resistant strains of *Staphylococcus aureus* and *Streptococcus pyogenes*, making it a viable first-line alternative in AMR-affected areas.

Our study has several strengths. First, we complied with the methodological guidelines for a high-quality systematic review and meta-analysis established by PRISMA 2020 [16]. Second, we systematically searched four databases and rigorously selected each study. Third, we used the random-effects model to generalize the results to different populations. Fourth, we performed a strict risk of bias assessment using the RoB 2.0 tool, ensuring the robustness of our findings. Finally, we used the GRADE methodology, providing certainty about the results.

Antibiotic resistance has spread worldwide, which has negatively impacted the treatment of patients with impetigo. Resistance to standard topical therapies such as mupirocin has been reported in several regions. The findings of this meta-analysis are relevant in the context of current clinical guidelines for impetigo management. The IDSA and NICE guidelines recommend topical antibiotics, such as mupirocin or fusidic acid, as first-line treatments for localized lesions, reserving oral antibiotics for more extensive cases. However, increasing resistance to mupirocins been reported, which may reduce its effectiveness in certain regions. The results of this study suggest that ozenoxacin 1% is comparable in efficacy to mupirocin but with potential advantages, such as faster bactericidal action and lower resistance rates. Given the low rate of adverse effects, ozenoxacin may be a viable alternative, especially in cases where mupirocin resistance is a concern. Future clinical trials comparing ozenoxacin directly with other topical agents will help to refine its place in treatment algorithms [18].

Colonization by *Staphylococcus aureus* is a key factor in the recurrence of impetigo and other skin infections. The primary portage areas include the nasal cavity, which is considered the main reservoir, with persistent colonization in 20–30% of individuals; the perineal and groin regions, which are frequently colonized, particularly in hospitalized or immunocompromised patients; the axillae and hands, which play a crucial role in bacterial transmission and auto-inoculation; and the oropharyngeal mucosa, which can serve as a colonization site in populations with recurrent infections. Recent studies suggest that ozenoxacin 1% cream is effective in reducing bacterial burden in localized skin infections, with a low risk of resistance development. While mupirocin is commonly used for the nasal decolonization of *S. aureus*, the increasing prevalence of mupirocin-resistant strains underscores the need for alternative topical agents. Given its rapid bactericidal action and favorable safety profile, ozenoxacin could be considered a promising option for targeted decolonization strategies in patients with recurrent infections or those at high risk of persistent colonization. Further research is needed to evaluate its efficacy in eradicating *S. aureus* from portage sites and its potential role in methicillin-resistant *Staphylococcus aureus* (MRSA) decolonization protocols.

The safety profile of ozenoxacin is an essential factor for its potential clinical use. In this meta-analysis, the incidence of adverse events was low, and most reactions were mild and self-limiting. This aligns with previous studies that have demonstrated a favorable tolerability profile, even in pediatric populations. Unlike systemic antibiotics, ozenoxacin is applied topically, which reduces the risk of systemic adverse effects. Given these findings, ozenoxacin appears to be a safe and effective option for treating impetigo. However, future studies with longer follow-ups and larger sample sizes should further assess its long-term safety profile.

These findings have important implications for clinical practice and public health. The clinical and bacteriological success of ozenoxacin 1% against impetigo supports the possibility of being considered a first-line alternative for treating impetigo, particularly in regions with a high prevalence and bacterial resistance.

From our study, we recognize the importance of generating more scientific evidence about the efficacy of ozenoxacin in combination with other treatments or other therapeutic alternatives. In addition, more extensive studies and cost-effectiveness evaluations are needed to determine the true impact of ozenoxacin in daily clinical practice.

One of the major challenges in antibiotic therapy is the development of bacterial resistance, which limits the long-term treatment efficacy. While ozenoxacin has demonstrated low resistance rates in clinical and in vitro studies, the possibility of resistance emergence cannot be ignored. Children under five years of age are disproportionately affected by impetigo, especially in tropical and low-resource settings, where access to healthcare is limited. Ozenoxacin’s topical application, rapid bactericidal action, and favorable safety profile make it particularly suitable for pediatric use. In immunocompromised or elderly patients, the risk of secondary bacterial infections and complications is higher. The ability of ozenoxacin to effectively eradicate bacterial colonization with minimal systemic absorption reduces the likelihood of systemic adverse effects. The inclusion of ozenoxacin in national and international treatment guidelines could help diversify available treatment options, reducing the dependence on high-resistance antibiotics. In resource-limited settings, the affordability and cost-effectiveness of ozenoxacin compared to emerging antibiotic-resistant therapies should be further evaluated. Ongoing surveillance programs and stewardship policies will be essential to monitor resistance trends and ensure sustainable antibiotic use.

Emerging evidence suggests that Bacillus species, particularly Bacillus simplex and Bacillus oleronius, play a role in the pathophysiology of rosacea, acting as endosymbionts of the Demodex folliculorum mite [19]. Bacillus oleronius has been shown to trigger proinflammatory responses, leading to increased skin inflammation, which contributes to rosacea’s pathogenesis. Other species, such as Bacillus cereus and Bacillus pumilus, have been implicated in opportunistic skin infections and may influence conditions like acne [19].

While ozenoxacin is primarily indicated for impetigo caused by *Staphylococcus aureus* and *Streptococcus pyogenes*, its broad-spectrum bactericidal activity against Gram-positive bacteria raises the possibility of activity against Bacillus species. Given its topical formulation and low resistance potential, ozenoxacin could be explored as a potential therapeutic option for rosacea and other skin conditions associated with Demodex-related bacterial overgrowth. Future studies should evaluate its efficacy in reducing Bacillus colonization in facial regions, particularly in patients with rosacea or acne-prone skin.

The main limitation of this systematic review is that it contains a small number of included studies (n = 3) due to the need for more research on treating impetigo compared to ozenoxacin, with a relatively recent appearance of the drug on the market. This contributes to significant heterogeneity between studies, making it difficult to compare results. On the other hand, according to the GRADE evaluation, it also decreases the certainty of the results and conclusions.

This study was a systematic review with meta-analysis. The Reference Items for Systematic Reviews and Meta-Analyses (PRISMA 2020) informed this review and was registered in PROSPERO (CRD42024578375). Data are provided within the manuscript or Supplementary Information Files [16].

## 5. Conclusions

In patients with impetigo, ozenoxacin 1% may significantly increase the clinical and microbiological success, and significantly reduce the microbiological clinical failure compared with standard treatment.

This meta-analysis provides clear evidence for the efficacy of ozenoxacin 1% in treating impetigo in pediatric and adult populations. Given its effectiveness and favorable safety profile, ozenoxacin should be considered a viable option for treating this dermatologic condition.

## Figures and Tables

**Table 1:** Characteristics of the included studies.

Author, Year	Country	Type and Phase	N^o^ Patients per Ozenoxacin Arm	N^o^ Patients per Placebo Arm	Age	Intervention			Control
						Drug	Doses	Description	Description
Gropper, 2014 [14]	South Africa, Germany, Romania, USA, Ukraine	Randomized controlled trial, phase III, multicenter, parallel	(155 patients); Males or females aged ≥ 2 years with a clinical diagnosis of bullous or non-bullous impetigo, a total skin infection rating scale (SIRS) score of at least 8, and a total affected area of 1–100 cm^2^ with surrounding erythema.	(156 patients)	E: 16.1 ± 17.71 *p*: 17.3 ± 17.18	Ozenoxacin 1% cream	Application twice daily for 5 days	Topical application of cream in the morning and evening on all areas affected by impetigo for 5 days (10 applications).	Placebo cream application
Rosen, 2018 [4]	USA, Russia, South Africa, Germany, Romania, Spain.	Randomized controlled trial, phase III, multicenter, parallel	(206 patients); Children aged 2 months or older, with a clinical diagnosis of impetigo and a total SIRS score of at least 3.	(206 patients)	18.6	Ozenoxacin 1% cream	Application of 0.5 g twice daily for 5 days	Topical application the size of the fingertip covering an area of 10 cm^2^ twice a day for 5 days.	Placebo cream application
Dash, 2023 [15]	India	Randomized controlled trial, phase III, single center, parallel	(16 patients); Children aged 2 months and 12 years who had a medical diagnosis of impetigo and obtained a minimum total score of 3 on the Skin Infection Rating Scale (SIRS).	(17 patients)	E: 5.6 *p*: 5.4	Ozenoxacin 1% cream	Application twice a daily for 7 days	Topical application twice daily for a duration of seven days	Topical application of mupirocin 2%

## Data Availability

Data are contained within the article.

## Supplementary Materials

The following supporting information can be downloaded at: https://www.mdpi.com/article/10.3390/jcm14072157/s1, Table S1: Strategy search; Table S2: GRADE assessment.
