# Supplementary material for: Ozenoxacin 1% in Pediatric and Adult Patients with Impetigo: A Meta-Analysis of Randomized Trials
Source: J Clin Med. 2025 Mar 21;14(7):2157. doi: 10.3390/jcm14072157 (PMC11989652; doi:10.3390/jcm14072157)
Supplement: Supplementary file 1 [file jcm-14-02157-s001.zip › jcm-3488921-supplementary.pdf]

Efficacy of ozenoxacin 1% in pediatric and adult patients with impetigo: A meta-analysis of randomized trials

SUPPLEMENTARY MATERIAL

Table S1. Search strategy on each databases

SEARCH ENGINE	STRATEGY	RESULTS
**PUBMED**	#1= (Child OR Children OR Adolescents OR Adolescence OR "Adolescents, Female" OR "Adolescent, Female" OR "Female Adolescent" OR "Female Adolescents" OR "Adolescents, Male" OR "Adolescent, Male" OR "Male Adolescent" OR "Male Adolescents" OR Youth OR Youths OR Teens OR Teen OR Teenagers OR Teenager)<br><br>#2= (Impetigo OR impetigos OR "impetigo, Contagiosa" OR "contagiosa, impetigo" OR "Contagiosas, impetigo" OR "Impetigo, contagiosas")<br><br>#3= (Ozenoxacin OR "1-cyclopropyl-8-methyl-7-(5-methyl-6-(methylamino)-3-pyridinyl)-4-oxo-1,4-dihydro-3- quinolinecarboxylic acid" OR ozanex OR xepi)	19
**SCOPUS**	(TITLE-ABS-KEY(Child OR Children OR Adolescents OR Adolescence OR "Adolescents, Female" OR "Adolescent, Female" OR "Female Adolescent" OR "Female Adolescents" OR "Adolescents, Male" OR "Adolescent, Male" OR "Male Adolescent" OR "Male Adolescents" OR Youth OR Youths OR Teens OR Teen OR Teenagers OR Teenager)) AND (TITLE-ABS-KEY(Impetigo OR impetigos OR "impetigo, Contagiosa" OR "contagiosa, impetigo" OR "Contagiosas, impetigo" OR "Impetigo, contagiosas")) AND (TITLE-ABS-KEY (Ozenoxacin OR "1-cyclopropyl-8-methyl-7-(5-methyl-6-(methylamino)-3-pyridinyl)-4-oxo-1,4-dihydro-3- quinolinecarboxylic acid" OR ozanex OR xepi))	27
**WEB OF SCIENCE**	#1= (Child OR Children OR Adolescents OR Adolescence OR "Adolescents, Female" OR "Adolescent, Female" OR "Female Adolescent" OR "Female Adolescents" OR "Adolescents, Male" OR "Adolescent, Male" OR "Male Adolescent" OR "Male Adolescents" OR Youth OR Youths OR Teens OR Teen OR Teenagers OR Teenager)<br><br>#2= (Impetigo OR impetigos OR "impetigo, Contagiosa" OR "contagiosa, impetigo" OR "Contagiosas, impetigo" OR "Impetigo, contagiosas")<br><br>#3= (Ozenoxacin OR "1-cyclopropyl-8-methyl-7-(5-methyl-6-(methylamino)-3-pyridinyl)-4-oxo-1,4-dihydro-3- quinolinecarboxylic acid" OR ozanex OR xepi)	11
**EMBASE**	#1= 'child'/exp OR 'child'<br>#2= 'ozenoxacin'<br>3= 'impetigo'/exp OR 'impetigo'	18

Table S2. GRADE Certainty of evidence

Outcomes	№ of participants (studies) Follow-up	Certainty of the evidence (GRADE)	Relative effect (95% CI)	Anticipated absolute effects	
				Risk with Standard of care	Risk difference with Ozenoxacyn
Clinical success (CS) assessed with: RR	756 (3 RCTs)	⊕⊕⊕◯ Moderate	**RR 1.14** (1.12 to 1.17)	763 per 1,000	**107 more per 1,000** (92 more to 130 more)
Clinical failure (CF) assessed with: RR	756 (3 RCTs)	⊕⊕⊕◯ Moderate	**RR 0.54** (0.39 to 0.75)	203 per 1,000	**93 fewer per 1,000** (124 fewer to 51 fewer)
Microbiological success (MS) assessed with: RR	583 (3 RCTs)	⊕⊕⊕◯ Moderate	**RR 1.28** (1.05 to 1.58)	649 per 1,000	**182 more per 1,000** (32 more to 377 more)
Microbiological Failure (MF) assessed with: RR	583 (3 RCTs)	⊕⊕⊕◯ Moderate	**RR 0.31** (0.21 to 0.46)	264 per 1,000	**182 fewer per 1,000** (208 fewer to 143 fewer)

*__The risk in the intervention group__ (and its 95% confidence interval) is based on the assumed risk in the comparison group and the **relative effect** of the intervention (and its 95% CI).

CI: confidence interval; **RR:** risk ratio

GRADE Working Group grades of evidence
High certainty: we are very confident that the true effect lies close to that of the estimate of the effect.
Moderate certainty: we are moderately confident in the effect estimate: the true effect is likely to be close to the estimate of the effect, but there is a possibility that it is substantially different.
Low certainty: our confidence in the effect estimate is limited: the true effect may be substantially different from the estimate of the effect.
Very low certainty: we have very little confidence in the effect estimate: the true effect is likely to be substantially different from the estimate of effect.
